# Unmet Needs for Support in Activities of Daily Living among Older Persons: The Effects of Family and Household Structures in a Low- and Middle-Income Context

**DOI:** 10.3390/geriatrics9010005

**Published:** 2024-01-03

**Authors:** Jacob Wale Mobolaji

**Affiliations:** Department of Demography and Social Statistics, Obafemi Awolowo University, Ile-Ife 220282, Nigeria; jmobolaji@oauife.edu.ng

**Keywords:** unmet needs, social support, activities of daily living, family/household structure, older person, Nigeria

## Abstract

The unmet need for assistance in activities of daily living (ADLs) accentuates older persons’ risk of falls, ill health, hospitalisation, and mortality. In Nigeria, the family arrangements through which older persons derive support are changing due to modernisation, migration, and economic challenges. How the family dynamics explain the unmet needs is poorly understood. This study investigates the influence of family and household structures on older persons’ unmet needs in ADLs in southwestern Nigeria. The study analysed the data of 827 older adults aged ≥65 years selected from Oyo State, southwestern Nigeria, using a multi-stage sampling design. Associations were examined using the Poisson–logit hurdle regression model. From the results, 65% of older persons with difficulties had unmet needs in instrumental ADLs and 59% in basic ADLs. Increased unmet needs were associated with older persons living with non-family members (β = 0.19; *p* < 0.01; 95% C.I. = 0.05–0.32) and widows (β = 0.27; *p* < 0.01; 95% C.I. = 0.13–0.42). Conversely, unmet needs decreased with higher family size (β = −0.06; *p* < 0.001; 95% C.I. = −0.08–−0.03), living in rich households (β = −0.29; *p* < 0.001; 95% C.I. = −0.42–−0.17), not being the household head (β = −0.27; *p* < 0.001; 95% C.I. = −0.40–−0.15), close family bonds, and proximity to children/caregivers. The study recommends alternative or complementary home-based support mechanisms for seniors with vulnerable family settings in southwestern Nigeria.

## 1. Introduction

Old age is sometimes characterised by emerging needs and difficulties in performing activities of daily living (ADLs) due to decreasing physical functioning and deteriorating health. Advanced age is sometimes marked by frailty and profound inability to live independently, leading to increased dependence on external support to maintain well-being [1,2]. Those who struggle to carry out essential ADLs are at risk of self-injury, domestic hazards, depression, and a diminished quality of life, as long as their needs remain unmet [3]. The term ADLs encompasses both basic activities of daily living (BADLs) and instrumental activities of daily living (IADLs) and is indicative of an individual’s ability to live independently [3].

BADLs include the ability to independently ambulate, eat, maintain personal hygiene, manage continence, and use the toilet appropriately [4]. In contrast, IADLs involve more complex activities such as the ability to independently manage telephone use, laundry, shopping, running errands, self-transport either by driving or taking public transport, meal preparation, medication management, housework, and financial management [5]. Functional limitations in these aspects of life pose significant challenges without external support.

The likelihood of individuals’ inability to live independently and their need for long-term support increases as they age. However, these trends can vary based on individuals’ socioeconomic conditions and the level of access to social support. Older persons in socioeconomically disadvantaged societies in low- and middle-income countries (LMICs), particularly in sub-Saharan Africa (SSA), with limited access to healthcare services and social support, are at higher risk of experiencing difficulties in ADLs, poor health outcomes, and shorter life expectancy compared with their counterparts in high-income societies [6]. These health outcomes combined with persistent economic and social challenges that hinder families from adequately supporting older family members lead to a greater caregiving burden in the region compared with other parts of the world. This, in turn, results in unmet needs for older individuals with ADL difficulties.

Unmet needs in ADLs occur when individuals with ADL difficulties receive no assistance or require additional support for at least one ADL [7]. If left unaddressed, these unmet needs can worsen an individual’s health condition, and increase the risk of falls, hospitalisation, and mortality among older people [8,9,10,11]. Existing evidence suggests that a significant proportion of older people worldwide experience unmet needs in their daily functioning, although most of these studies have been conducted in high-income contexts. For example, a study in Quebec, Canada revealed that over one-quarter of older persons aged 75 years or older had unmet needs in ADLs [12]. Studies from other countries, such as the United States [8,13] and the United Kingdom [14] reported a similar prevalence of unmet needs, ranging from 20% to 50% among older persons. A more recent study in the United Kingdom differentiated between BADLs and IADLs, reporting a 55% prevalence of unmet needs in BADLs and 24% in IADLs [15]. Given the substantial number of older persons affected, the adverse outcomes associated with unmet needs could lead to increased healthcare expenditure, strain on available caregiving facilities, and a reduction in the quality of healthcare services. Therefore, it is imperative to estimate unmet needs in ADLs among older adults in LMICs to identify those in need of assistance or additional support to maintain their safety, health, and quality of life. This also signals the future demand for long-term care services.

In SSA, families continue to serve as the primary safety net for older persons to access care and lead healthy lives [16,17]. For example, in southwestern Nigeria, older individuals rely heavily on family members for support due to the absence of institutionalised social support systems and the limited availability of, as well as the high cost of, caregiving homes. Traditional African family structures further strengthen family support, with members providing emotional, domestic, economic, and various other forms of support to their older relatives. However, evolving family structures influenced by modernisation, which promotes nuclear family settings and shifts caregiving responsibilities to adult children of older individuals [18], are challenging this family support system. Support from adult children is also declining due to economic constraints, changing social dynamics and the migration of adult children, all of which have altered the region’s traditional living arrangements for older adults [19]. The impact of these changes on older adults, especially those with ADL difficulties, remains poorly understood. In 2006, Gureje and colleagues reported a 20% prevalence of unmet needs in ADLs in some communities of southwestern Nigeria [2]. As the region continues to experience modernisation, it is crucial to monitor the current situation and assess how changes in family dynamics have contributed to unmet needs.

Furthermore, various economic and social factors can constrain the provision of support to older family members. These factors include the physical capacity of caregivers, time constraints, proximity to the recipient, and the availability of family members [20,21,22]. Caregivers with full-time employment may find it difficult to support older individuals due to time constraints [23,24]. Financial limitations may also hinder their ability to afford the costs of travelling and providing medium- or long-term support [25], and the physical challenges faced by caregivers due to health issues can limit their capacity to provide support. While additional support may be offered by other available social networks, it may not be appropriate or adequate for ADL support [26,27,28].

In traditional African family settings, older individuals are often considered family heads, and evidence suggests that holding this role can be beneficial, as it grants them access to control over household resources [29]. Family heads typically receive both physical and emotional support from family members. Those living with dependent children or grandchildren can receive ADL support from their offspring. However, due to changing living arrangements and the increasing number of older persons living alone, the avenues for support from household members are diminishing. Additionally, being a household head may be more challenging than advantageous for some older adults, especially in Nigeria, where the majority of older persons are vulnerable and struggle to meet their daily financial, nutritional, and healthcare needs [30]. It is essential to explore the impact of being a household head on the well-being of older individuals in resource-constrained settings like Nigeria.

Given the unstable socioeconomic situation of caregivers, changing African traditional family structures in SSA due to modernisation, migration, the impact of diseases like AIDS, and the mortality of the younger generation [19,31], there are critical questions that require empirical answers concerning the unmet needs in ADLs among older persons in the region. How do current household structures such as family size, family type, proximity to children or caregivers, and the living arrangements of older persons affect their unmet needs in ADLs? This study aims to investigate unmet needs in ADLs and examine how family and household structures contribute to the unmet needs among older people in southwestern Nigeria.

This study utilises the framework of the informal care model (ICM), which posits that the provision of support by caregivers is influenced by societal norms of family responsibility towards older members [32,33]. According to the model, the provision of support for an older person is driven by the caregiver’s sense of societal responsibility, religious beliefs, the quality of the relationship with the recipient, and inherent challenges. However, the flow of such support may encounter constraints, including geographical distance, financial limitations, time constraints, physical capacity, and the circumstances of the caregiver. The provision of family support is rooted in the type and strength of relationships, especially in the child–parent relationship, where affection and cohesion motivate children to support their parents [34]. Relationships characterised by love, strong bonds, and regular interaction encourage individuals to provide support [20]. Nevertheless, the unstable economic conditions of families and social changes in LMICs may impact these relationships, constrict the channels through which support reaches older persons, and exacerbate their unmet needs, particularly in ADLs. This model is being examined in the context of sub-Saharan African settings to gain further insights into its applicability to diverse socioeconomic environments.

## 2. Materials and Methods

### 2.1. Study Design and Data Source

The study utilised quantitative data from older persons aged 65 years and above in Oyo State, southwestern Nigeria. The state’s population is estimated at 7,976,081 people, including 478,118 older persons, which is the second-highest in the region after Lagos. The proportion of older persons (6.2%) among the total male population of the state was higher compared to their proportion among the female population (5.8%), unlike in other states [35]. This gender disparity, in addition to the substantial older population in the state, justified the selection of this location for this study. While this gender disparity suggests a potential lower survival rate for older women relative to men, a lower sex ratio, or an increased male-child mortality rate, the exact reasons require empirical evidence.

The study adopted a proportional sampling approach, as described by Casagrande, Fleiss, and their associates [36,37], and included 394 men and 433 women, resulting in a total sample size of 827 older adults. This approach was used to account for the gender disproportionality in access to social support in later life, which had been established in a prior study [19]. The sample size was determined based on a 5% level of significance (α) and 90% statistical power (β). The sample size is sufficiently large to achieve statistical significance with a minimum effect size of d = 1.3. This implies that there was a 90% probability of correctly accepting the alternative hypotheses. Furthermore, the sample size used in this study is 120% larger than the sample size of 375 used in the Demographic and Health Survey 2018 [38] for the study area. Given these considerations, the sample size for this study is considered adequate and representative.

The study sample was selected using a multistage cluster design. Oyo State is divided into three senatorial districts (Oyo Central, North, and South), containing 13, 11, and 9 local government areas (LGAs), respectively. The LGAs were categorised into rural and urban strata based on population size and available social infrastructure. One LGA from the rural and one from the urban stratum were selected from each senatorial district. Clusters were systematically chosen from the sampled LGAs using existing enumeration area (EA) maps of the state. Eligible respondents from the randomly selected households in each EA of the selected clusters were interviewed for this study. A well-structured and interviewer-administered questionnaire was used to collect relevant information related to the respondents’ sociodemographic characteristics, areas of daily living in which they have experienced difficulties, and the types and extent of support they received in those areas of difficulties.

I recruited and trained ten research assistants along with three supervisors to assist in the electronic collection of research data using Android devices. The supervisors provided oversight and guidance to ensure data quality. The data collected were monitored in real time, allowing for immediate attention and correction by research assistants. All data were collected using Research Electronic Data Capture (REDCap), a secured web-based data collection software used for collecting and securely storing research data [39,40]. Subsequently, the collected data were exported in Stata format.

### 2.2. Variables and Measures

The dependent variable in this study is the experience of unmet needs for support in ADLs by older adults. ADLs encompass both basic and instrumental activities. To assess unmet needs, older adults were asked about the extent of their difficulties or needs in the following ADLs over the previous 30 days: managing their finances, eating independently, taking care of personal appearance, dressing and undressing, getting out of bed, reaching the toilet on time, bathing independently, travelling to places beyond walking distance, fetching water, going to the market for shopping, performing housework, preparing their own meals, walking two kilometres, leaving the house, crossing the road, and managing their medications. The extent of difficulty was measured on a 3-point scale: 0 for no difficulty/need, 1 for moderate difficulty, and 2 for severe difficulty. Respondents were also asked about the extent of receiving support in these ADL items over the last 30 days, with the responses categorised as adequate support (coded 0), limited support (coded 1), or no support (coded 2).

Combining the questions regarding the extent of ADL difficulties and support received, respondents who had no difficulties or those with difficulties but who received adequate support were categorised as having no unmet need (code = 0). Meanwhile, respondents who experienced either moderate or severe difficulties but received limited or no support were categorised as having unmet need (code = 1) for each of the items. Respondents were considered as having unmet needs if they faced difficulty in one or more of the ADL items but received little or no support in those areas.

The explanatory variables in this study, informed by the existing literature [15,17,29,41,42], encompass family and household structures, including family size (number of living children), current marital status, household wealth status (low, moderate, high), family type (monogamy or polygamy), household living arrangements (living alone, with immediate family, with others), household headship (self, spouse, son/daughter, others), proximity to children/caregivers (far, somewhat close, close), caregivers’ socioeconomic status (low, moderate, high) and family bond (not close, somewhat close, very close). Additionally, other explanatory variables operationalised as covariates include age, level of education, religion, gender, rural–urban place of residence, participation in any economic activity, and sources of income.

### 2.3. Statistical Analysis

The data were analysed at univariate, bivariate, and multivariable levels. At the univariate level, the proportion of older persons experiencing difficulties and unmet needs in both BADLs and IADLs was compared across different gender and household/family characteristics using percentage distribution. Bivariate associations between the experience of unmet needs and household/family variables were examined using the Chi-square test of independence.

To estimate the effect of household/family structures on the probability of experiencing unmet needs and the number of unmet needs in later life, a Poisson–logit hurdle regression model was employed. The hurdle model is a two-stage count model, with a binomial process generating zeros and otherwise in the first stage. In the second stage, the non-zero positive counts indicating zero-truncated distributions are modelled [43,44,45]. The number of zero unmet needs in the data was inflated by the number of older persons with no difficulty in any of the ADLs, suggesting the need for a zero-inflated regression model. Since the Poisson–logit hurdle regression model can handle both zero-inflated and zero-deflated data [46] and the diagnostic analysis indicates the absence of overdispersion, a critical assumption for Poisson regression, the Poisson–logit hurdle model is the most appropriate for this study. It has been used in previous studies investigating the factors affecting health and behavioural outcomes [47].

Given that $Y_{j}$ is a random variable representing the response of the *j*th observation, *j* = 1, 2, …, m, where m is the total number of observations, and the values are non-negative with a significant frequency of zero values ($Y_{j}$ = 0) leading to a skewed distribution towards zero, the count distribution of the outcome variable y follows a Poisson distribution; the zero-truncated Poisson model is expressed as:

(1) $$P\left(Y_{j}=y_{j}\right)=\left\{\begin{array}{c} \;\;\;w_{j}\;\;\;\;\;\;\;\;\;\;\;\;\;\;\;\;\;\;\;\;\;;y_{j}=0\;\\ \left(1-w_{j}\right)\;\frac{e^{-\mu_{j}}{\mu_{j}}^{y_{j}}}{y_{j}!(1-e^{-\mu_{j}})}\;{;y}_{j}>0 \end{array}\right.$$

where $w_{j}$ is the probability of an observation being a zero component, *y_i_* = 0 is the distribution of the outcome at zero, and $y_{j}$ is the count distribution for $y_{j}$ > 0. Since the covariates can influence the probability of zero $w_{j}$, as in the zero-inflated model, and $\mu_{j}$ is the mean for a hurdle model, the hurdle model is expressed as follows:

(2) $$\mathrm{Logit}\;(w_{j})={u_{j}}^{\tau }\beta_{j}\;\mathrm{and}\;\mathrm{Logit}\;(\mu_{j})={v_{j}}^{\tau }\lambda_{j}$$

where $u_{j}=1,u_{1},u_{2},\ldots ,u_{n}$ and $v_{j}=1,v_{1},v_{2},\ldots ,v_{n}$ are the *j*th row dimensional vector of the covariates, $\beta_{j}={\beta_{0},\beta }_{1},\beta_{2},\ldots ,\beta_{n}$ and $\lambda_{j}=\lambda_{0},\lambda_{1},\lambda_{2},\ldots ,\lambda_{n}$ are the regression coefficients for $u_{j}$ and $v_{j}$ covariates. 

The Poisson–logit estimate was based on maximum likelihood estimation (MLE), which is the regular estimation method for a count model. The estimates of Pearson’s Chi-square statistics, log likelihood, and Akaike’s information criteria (AIC) were used to assess the model’s performance and indicated that the model is a good fit [43]. All estimates of the model were based on a 95% confidence interval. The data were analysed using Stata, version 15.1.

Before the multivariable analysis, the multicollinearity test was conducted on the main explanatory variables using the variance inflation factor (VIF). The result indicated that the VIFs for all the variables were below 5, which is the recommended threshold for multicollinearity [48,49]. Therefore, there was no multicollinearity among the variables (see the Appendix A). Additionally, the multivariable analysis adjusted for the covariates as listed above. However, the results of the covariates are not included in the tables presented within the manuscript to avoid overly lengthy and complex tables. See the Appendix A for details about the results of the covariates.

### 2.4. Ethical Approval

This study was conducted in accordance with the guidelines of the Declaration of Helsinki and approved by the Health Research Ethics Committee (HREC) of the Institute of Public Health, Obafemi Awolowo University, Ile-Ife with HREC No: IPHOAU/12/1330. Approval was granted on 5 August, 2019. The data were collected within the 12-month time frame given by the committee.

## 3. Results

The distributions of the study sample across various sociodemographic and socioeconomic characteristics are presented in Table 1. Generally, approximately two-thirds (66%) of the study participants were aged 70 years or older, although some gender disparities were observed, with a higher proportion of women in the advanced age group aged 80 or above (32%) compared with men (26%). About 48% of the respondents were currently married, 43% were widowed, and the remainder had never been married, separated, or divorced. However, a male–female disproportion was evident, with only 29% of women currently married compared with 69% of men. This lower proportion of currently married women translated to a higher proportion of widows among them (66%) compared with men (19%).

Approximately three-fifths of the respondents, regardless of gender, identified as Christians (62%). Nearly half (49%) had no formal education, with 33% among men and 63% among women, reflecting the long-standing gender inequality in education in Africa. Additionally, two-thirds (66%) were engaged in economic activities, with a significant proportion (29%) involved in petty trading (45% for women and 11% for men) or farming (19%) (28% for men and 10% for women, respectively).

For more than half (58%) of the sampled older persons in the study area, personal work or pension was their primary source of income, while about one-third (25% for men and 41% for women) relied mainly on their children as an income source. This gender disparity in income sources suggests that financial support from children is directed more toward older women than men.

### 3.1. Difficulties in ADL and Corresponding Lack of Support among Older Adults

The analysis in this study revealed that about 59% and 31% of the sampled older persons in Oyo State experienced one or more difficulties with BADLs and IADLs, respectively (see Table 2). Among the ADL difficulties, getting to places beyond walking distance (47%), fetching water (40%), and going shopping (34%) were the most frequently reported IADLs difficulties. On the other hand, for BADL difficulties, walking about 2 km (23%) and getting out of the house or crossing the road (22%) were the most common. Handling their own money (18%) and bathing themselves (14%) were the least reported IADL and BADL difficulties, respectively. Among respondents who reported difficulties in ADLs, 47–67% had unmet needs. These patterns varied across genders, with a higher share of unmet needs among older women (53–78%) compared with men (39–61%).

### 3.2. Patterns of Unmet Needs in BADLs and IADLs among Older Adults

**Figure 1:**
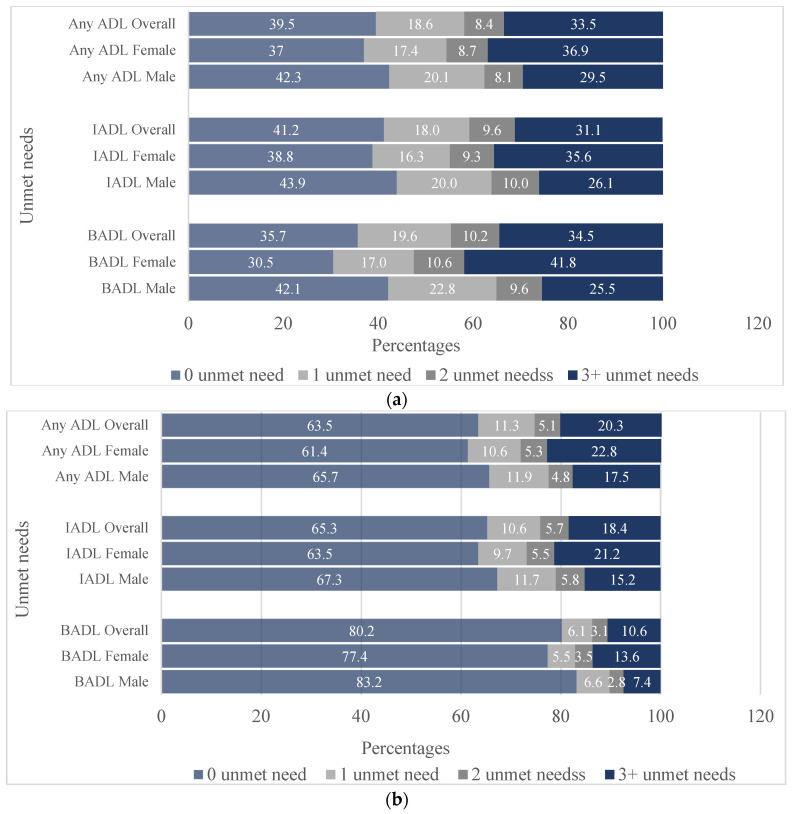
Distribution of unmet needs in ADLs among older adults in Oyo State. (**a**) Distribution of unmet needs based on the respondents with difficulties. (**b**) Distribution of unmet needs based on the total study sample.

The results show that difficulties in IADLs were more prevalent than in BADLs for older people in the study area. Similarly, the prevalence of unmet needs in IADLs among those with difficulties was higher than that in BADLs (see Figure 1a). This disparity may be because many people who have difficulty in IADLs are still able to perform some BADLs tasks independently without difficulty. Of the respondents who had difficulty in any of the activities of daily living, about one-third (31%) had three or more unmet needs in IADLs, with a higher proportion among women (36%) compared with men (26%). The pattern was similar for BADLs, with a higher proportion among women (42%) than men (26%). Based on the total sample of the respondents, about 18% and 11% had three or more unmet needs in IADLs and BADLs, respectively (see Figure 1b).

Though functional abilities decrease as individuals age, the availability and adequacy of support mechanisms determine the timing and level of unmet needs in an older person. The overall unmet needs of the respondents in this study followed a declining pattern with age. Older men and women in the age group 65–69 both had the highest share of individuals with one or more unmet needs compared with their counterparts in the oldest age group, 85 years or above, who had the lowest share (result not shown). With this level of prevalence at ages 65–69 years, early onset of unmet needs may lower the quality of life and the chance of longevity for persons of this age group.

### 3.3. Family/Household Structures and Unmet Needs of Older Persons

The results in Table 3 show the relationship between the selected family structures of older adults and their experience of unmet needs in activities of daily living. Having children to lean on for support is highly beneficial in later life, especially in resource-constrained societies where formal support is limited. In this study, the share of older adults with unmet needs in BADLs was higher for those with smaller (≤4) family sizes (25%) compared with those with five or more family members (16%). Household living arrangements also play vital roles in accessing household support. A higher proportion of respondents who were living alone (25%) or with others other than immediate family (24%) experienced more unmet needs than those living with a spouse and/or children (16%). Similarly, older persons who were household heads reported a higher prevalence of unmet needs in BADLs (24%) compared with those who lived in households headed by someone else (13%). This variation is more noticeable among older women (29% versus 14%) than their male counterparts (19% versus 10%). In addition, the distance of older adults to their children potentially influences the frequency of visits and length of stay with their parent(s). Those who lived very close to their immediate family, especially their children, had a lower prevalence of unmet needs in BADLs (14%) compared with those who were not very close to their family (21–25%).

Unmet needs in IADLs, on the other hand, varied more with family size, living arrangements, and proximity to immediate family than with any other family/household attribute. Those with smaller (≤4) family sizes were 27 percentage points more likely to have unmet needs in IADLs than those with five or more family members (41% versus 30%). This disparity was more pronounced among women, with a 40% higher prevalence of unmet needs in IADLs (45% versus 27%) compared with men, with a six per cent difference (34% versus 32%). Similarly, respondents living either alone or with others had a 21–26% higher share of individuals with unmet needs than their counterparts living with their immediate family. Moreover, those who lived far away from or somewhat close to their immediate family had a 17–27% higher share of individuals with unmet needs in IADLs than those living very close to them. This pattern was consistent for women, unlike for men. Examining any of the unmet needs, either BADLs or IADLs, all the selected household attributes followed similar patterns and associations with BADLs except the household headship.

### 3.4. Family/Household Structures and Probability of Experiencing Unmet Needs in ADL—The Zero-Hurdle Model

The estimates of the zero hurdle (logit) model in the first panel of Table 4 indicate that the household wealth status, household headship, and proximity to children were significant determinants of the probability of experiencing unmet needs in BADLs among the respondents. The log odds of experiencing unmet needs in BADLs for older persons in households with moderate wealth status were 0.54 lower than those of poor wealth status (β = −0.54; *p* < 0.05; 95% C.I.= −1.04–−0.04). Conversely, the log odds were higher by 0.68 for older persons living in households headed by someone else (β = 0.68; *p* < 0.01; 95% C.I. = 0.22–1.14), and by 0.46 for those living close to their children (β = 0.46; *p* < 0.01; 95% C.I. = 0.04–0.88), holding all other factors constant. None of the family/household factors contributed to unmet needs in IADLs among the respondents in the study area.

### 3.5. Family/Household Structures and Counts of Unmet Needs in ADLs—The Zero-Truncated Poisson Regression Model

The adjusted estimates of the zero-truncated Poisson regression model, which is the count model which is the second part of the Poisson–logit hurdle model (as presented in the second panel of Table 4), elucidate the family and household factors contributing to changes in the number of unmet needs among respondents. Family size, household wealth status, family type, living arrangements, and family bond emerged as significant predictors of these changes. For older adults with difficulties in BADLs, the log count of unmet needs decreased by 0.04 with each unit increase in family size (β = −0.04; *p* < 0.05; 95% C.I. = −0.08–−0.001). Similarly, the log count decreased for those in wealthier households (β = −0.33; *p* < 0.01; 95% C.I.= −0.54–−0.12) and for those with very close family bonds (β = −0.26; *p* < 0.05; 95% C.I. = −0.49–−0.03) by 0.33 and 0.26, respectively, while holding all other factors constant. Conversely, the log count of unmet needs among those in polygamous families (β = 0.19; *p* < 0.05; 95% C.I. = 0.01–0.37) was 0.19 higher than that of those in monogamous families. For older individuals living with others, the log count of unmet needs was 0.31 higher (β = 0.31; *p* < 0.01; 95% C.I. = 0.069–0.52) than for those living alone.

In the case of IADLs, in addition to the household wealth status and family bonds, which maintained similar relationships with unmet needs as observed in BADLs, other factors that showed significant relationships with unmet needs in IADLs were household headship, caregivers’ socioeconomic status, proximity to immediate family, and current marital status. The log count of unmet needs decreased by 0.22 for older individuals living in households headed by someone else and for those living close to their caregivers/children. Conversely, widows and individuals with caregivers of moderate socioeconomic status had an increased log count of unmet needs in IADLs compared with married individuals and those with caregivers of lower socioeconomic status. The predictors of experiencing unmet needs in any ADLs are consistent with those identified for BADLs and IADLs.

## 4. Discussion

This study delves into the unmet needs of older persons in their activities of daily living, shedding light on the risk factors stemming from family and household dynamics. Understanding these factors is pivotal for designing appropriate interventions aimed at bolstering support for older individuals, particularly in a resource-limited setting like southwestern Nigeria.

This study, encompassing the entire sample of older adults, irrespective of their ability to perform ADLs, reveals that approximately 37% of older adults have unmet needs in one or more ADLs. Notably, the prevalence of unmet needs in IADLs stands at 35%, surpassing that in BADLs at 20%. When this prevalence is extrapolated to the population of older persons in the study area, it becomes evident that several thousands of older persons face unmet needs. This prevalence diverges from the 20% reported by Gureje and colleagues [2], possibly due to methodological differences in measuring and estimating the unmet needs. This disparity may also signal an upsurge in the prevalence of unmet needs. The current prevalence underscores the increasing vulnerability of older adults in southwestern Nigeria to deprivation and neglect. The situation may be attributed to the worsening economic conditions and the burgeoning population of older adults in Nigeria, which could strain the available resources. Adult children, often unemployed or facing job loss due to economic crisis, may lack the financial means for extended visits, caregiving, and financial support, contributing to a growing burden of unmet needs among older persons as their population swells without corresponding socioeconomic development.

This study further found that among older adults with difficulties in BADLs, about two-thirds experienced unmet needs, compared with about three-fifths of those with IADL difficulties. This finding demonstrates a higher prevalence of unmet needs than Vlachantoni’s (2019) UK study, which reported that 55% of older adults with difficulty in basic ADLs had unmet needs [15], along with 24% for those with challenges in instrumental ADLs. This disparity can be associated with the lower socioeconomic situation and limited informal support system for older persons in Nigeria compared with the UK. It is well documented that older adults in resource-constrained societies in many SSA countries are more vulnerable to a reduced quality of life compared with their counterparts in developed regions [2]. The adverse socioeconomic conditions constrain caregivers’ capacity to provide support to their older family members. Moreover, changes in social structure and traditional family settings due to modernization and adult children’s migration [19,50] also contribute to the insufficient or lack of support for older persons with ADL difficulties in southwestern Nigeria.

The unmet needs of the study participants diminish with age, a trend aligning with Vlachantoni’s findings in the UK [15]. While ADL difficulties increase with age, the declining unmet needs with age indicate a shortage or inadequacy of support received by the study sample. This suggests that individuals at an early stage of old age (65–69 or 60–69 years) may experience more unmet needs because caregivers may assume they are relatively young, strong, and without functional difficulties. Also, due to the increasing age at marriage, individuals in this lower age group are likely to have younger children who are still in school or unemployed. Consequently, their children may not be available or possess the financial capacity to provide all needed support.

From a gender perspective, the findings show that unmet needs among older women were higher compared with men at the bivariate level of analysis. Although this disparity did not hold at multivariable analysis level (see Appendix A), it aligns with observations by other scholars such as Akinyemi and Akinlo, who noted a higher unmet need for daily care and personal visits among older men in southwestern Nigeria compared with women [19]. Vlachantoni also reported a higher unmet need for ADLs among older men in the UK [15]. This could be explained by the fact that children tend to provide more support to their mothers than to their fathers [51,52]. However, the higher unmet need among men may not be linked solely to limited family support but also to the preservation of self-independence and dignity among men in patriarchal societies [28]. In the modern African family setting, as shown in this study, support in old age is predominantly sourced from immediate family members, including children and spouse. For older women, particularly widows, support may be limited due to the absence of spousal support.

Family and household structures, especially household wealth status, current marital status, family size, household living arrangements, family type, family bond, proximity to children/caregivers, children’s socioeconomic status, and household headship all play pivotal roles in addressing the needs of older people. In this study, although marital status does not determine the likelihood of having unmet needs, it significantly influences the number of unmet needs in IADLs. Widows or widowers are more likely to experience a higher number of unmet needs compared with those who are married. This indicates the importance of spousal support in long-term care for individuals with functional difficulties. Spouses often cohabit with their partners and provide more intensive and consistent domestic support compared with children, who often live away from their parents [53]. This is a crucial benefit that may elude those who are not currently married, as implied by this study.

Household wealth status, reflecting economic strength, plays a significant role in people’s well-being. The study found that older persons in households with moderate wealth status have a lower likelihood of experiencing unmet needs compared with those in households with poor wealth status. This aligns with the existing literature, suggesting that socioeconomic status can impact access to healthcare and support services [2,19]. Older individuals in wealthier households are more likely to have educated and economically stable children who can afford to hire paid caregivers and in-home services for their parents. Conversely, economically disadvantaged individuals face more barriers in obtaining assistance for their ADLs.

In old age, children, in addition to the spouse, are the primary caregivers. Hence, the number of adult children is a crucial family attribute that influences an older person’s access to support. In this study, a large family size is associated with a reduced number of unmet needs. This finding is consistent with Akinyemi’s observation that parents with more adult children received more support than their counterparts with fewer children [17]. Having more children increases the likelihood of an older person accessing support from multiple sources, particularly for ADL difficulties. Furthermore, the burden of caregiving is lighter when shared among children, although it can be financially burdensome for a smaller number of children, especially amidst economic downturn, time constraints, personal family responsibilities, and job demands [54].

However, it is essential to recognize that the composition and dynamics of the family are vital in accessing support, especially in terms of family bonds and children’s socioeconomic status. This study found that respondents with children of moderate socioeconomic status had a higher likelihood of experiencing unmet needs in IADLs. This may be linked to an increasing trend of migration among adult children whose financial capacity may not allow them to hire paid caregivers for their parents. Nonetheless, this study highlighted that a close family bond reduces the risk of increased unmet needs for older adults. The findings underscore the interconnectedness of support networks within families, emphasising the importance of not only focusing on the older person but also considering the capacity and willingness of their children or caregivers to provide support.

The influence of household headship on unmet needs is mixed. On one hand, living in a household headed by someone else, regardless of gender, is associated with an increased probability of having unmet needs in BADLs compared with living in a household headed by an older person. On the other hand, it is associated with a reduced number of unmet needs for an older person. This finding indicates that household headship has its advantages and disadvantages. Although a household head has some authority over household members and resource allocation [28], which could be beneficial for an agile older person when resources are available, it also comes with the mental stress and financial burden of meeting the needs of household members. Furthermore, it may not benefit older women who are often marginalised in household headship and resource sharing in a patriarchal society [55,56]. The challenges associated with household headship may negatively impact the mental and physical abilities of older individuals and increase their functional difficulties, to which caregivers may lack the capacity to respond adequately. 

The study further found that living with non-family members was associated with an increased number of unmet needs in BADLs and all ADLs combined. This association may be linked to the existing evidence that while living alone among older adults is on the increase and affects their well-being [28], living with family members strengthens domestic support and resource sharing among family or network members. This finding is also consistent with that of Wang and colleagues, who argued that older Chinese living with family members cope better with ADLs and recover faster from illness than those living with non-family members [57]. The nature of ADL needs requires long-term care, which non-family members may not be inclined to provide unless as paid services.

This study found that older persons living close to their children had a higher likelihood of experiencing unmet needs in BADLs. This counterintuitive finding may be linked to cultural and social factors specific to the study area in Nigeria. In some cases, proximity to children may not necessarily translate to increased support, and further investigation is needed to understand the underlying dynamics. This finding emphasises the importance of considering cultural and regional factors when designing support programmes.

One of the major strengths of this study is its use of quantitative data obtained from a representative sample of older persons selected using an appropriate probability sampling technique. Therefore, the findings can be generalised to the older population in the study area. However, the findings were based on cross-sectional data which cannot be used to determine causality. This does not compromise the validity of the findings, as associations from cross-sectional data do not imply causality but indicate plausible causality that should be explored through existing evidence and relevant theory [58]. Additionally, the analysis was based on self-reported information from respondents. While the data were meticulously collected by trained researchers, there was no other mechanism to validate the reported responses other than the probe questions used by the interviewers. Furthermore, older persons with dementia or chronic conditions were excluded from the data even though they may have access to better support.

## 5. Conclusions

In conclusion, this study identifies widowhood, living with non-family members, and living in a polygamous family are factors associated with increased unmet needs in ADL. Conversely, larger family sizes, close family bonds, and living in wealthier households headed by someone else other than the older adult reduce the risk of unmet needs in southwestern Nigeria. Intervention should focus on creating an enabling environment for families to support their older members. In addition, the need to look beyond family support in addressing the needs of older adults in southwestern Nigeria is apparent. As traditional household settings evolve, and older adults face modern transformation, social welfare provisions should be made for older adults to access formal long-term care at a subsidised rate.

## Figures and Tables

**Table 1:** Sociodemographic and socioeconomic characteristics of older adults in Oyo State.

Sociodemographic Characteristics	Male n = 394	Female n = 433	Both N = 827
Age group	n (%)	n (%)	n (%)
65–69	131 (33.3)	147 (34.0)	278 (33.6)
70–74	90 (22.8)	89 (20.5)	179 (21.6)
75–79	69 (17.5)	58 (13.4)	127 (15.4)
80–84	54 (13.7)	74 (17.1)	128 (15.5)
85+	50 (12.7)	65 (15.0)	115 (13.9)
Marital status			
Married, living with a spouse	272 (69.0)	124 (28.6)	396 (47.9)
Never married/separated/divorced	48 (12.2)	23 (5.3)	71 (8.6)
Widowed	74 (18.8)	286 (66.1)	360 (43.5)
Religion			
Christianity	238 (60.4)	278 (64.2)	516 (62.4)
Islam	149 (37.8)	154 (35.6)	303 (36.6)
Traditional	7 (1.8)	1 (0.2)	8 (1.0)
Level of education			
None	129 (32.7)	272 (62.8)	401 (48.5)
Primary	168 (42.6)	118 (27.3)	286 (34.6)
Secondary/higher	97 (24.7)	43 (9.9)	140 (16.9)
Place of residence			
Rural	165 (41.9)	191 (44.1)	356 (43.0)
Urban	229 (58.1)	242 (55.9)	471 (57.0)
Occupational status			
Not working ^a^	132 (33.5)	152 (35.1)	284(34.3)
Working	262 (66.5)	281 (64.9)	543 (65.7)
Type of economic activities			
Nothing/unemployed	106 (26.9)	147 (33.9)	253 (30.7)
Professional (e.g., doctor, lawyer, etc.)	5 (1.3)	1 (0.2)	6 (0.7)
Artisan	50 (12.7)	8 (1.9)	58 (7.0)
Business (large/medium scale)	22 (5.6)	27 (6.2)	49 (5.9)
Petty trading	44 (11.2)	193 (44.6)	237 (28.7)
Farming	110 (27.9)	45 (10.4)	155 (18.7)
Driving	15 (3.8)	0 (0.0)	15 (1.8)
Retiree	26 (6.6)	5 (1.1)	31 (3.7)
Others	16 (4.1)	7 (1.6)	23 (2.8)
Sources of income			
Self-employment/pension	260 (66.0)	223 (51.5)	483 (58.4)
Children/family	97 (24.6)	177 (40.9)	274 (33.1)
Both self and family	37 (9.4)	33 (7.6)	70 (8.5)
Total	394 (47.6)	433 (52.4)	827 (100.0)

^a^ Those doing nothing and retirees.

**Table 2:** Difficulties in ADLs and corresponding unmet needs among older persons in Oyo State.

ADL Items	Proportion with Difficulties			Proportion with Unmet Needs ^a^		
	Overall (N = 827)	Males (n = 394)	Females (n = 433)	Overall (N = 298)	Males (n = 113)	Females (n = 140)
	n (%)	n (%)	n (%)	n (%)	n (%)	n (%)
BADL items						
Eating on your own	143 (17.3)	64 (16.2)	79 (18.2)	95 (66.4)	37 (57.8)	58 (73.5)
Dressing and undressing self	117 (14.1)	49 (12.4)	68 (15.7)	78 (66.7)	25 (51.0)	53 (78.0)
Taking care of own appearance	124 (15.0)	53 (13.5)	71 (16.4)	78 (62.9)	26 (49.1)	52 (73.3)
Getting out of the house/crossing the road	185 (22.4)	77 (19.5)	108 (24.9)	120 (64.8)	44 (57.1)	76 (70.4)
Walking 2 km	189 (22.9)	83 (21.1)	106 (24.5)	119 (63.0)	44 (53.0)	75 (70.8)
Getting out of bed	116 (14.0)	48 (12.2)	68 (15.7)	78 (67.3)	27 (57.2)	51 (70.5)
Bathing yourself	112 (13.5)	51 (12.9)	61 (14.1)	71 (63.4)	27 (53.0)	44 (72.2)
Getting to the toilet on time	114 (13.8)	52 (13.2)	62 (14.3)	71 (64.3)	27 (51.9)	44 (71.0)
At least one BADL difficulty	253 (30.6)	113 (28.7)	140 (32.3)	193 (64.8)	66 (58.4)	98 (70.0)
IADL items						
Getting to places beyond walking distance	388 (46.9)	180 (45.7)	208 (48.0)	240 (61.8)	109 (60.6)	131 (63.0)
Going to market/for shopping	282 (34.0)	117 (29.7)	165 (38.1)	173 (61.4)	69 (59.0)	104 (63.1)
Preparing own meal	253 (30.6)	126 (32.0)	127 (29.3)	126 (49.8)	49 (38.9)	77 (60.7)
Taking your drugs and medication	150 (18.1)	67 (17.0)	83 (19.2)	92 (61.3)	35 (52.3)	57 (68.6)
Fetching water for yourself	330 (39.9)	151 (38.3)	179 (41.3)	154 (46.7)	59 (39.1)	95 (53.0)
Doing housework	254 (30.7)	109 (27.7)	145 (33.5)	138 (54.4)	53 (48.6)	85 (58.7)
Handling own money	147 (17.8)	60 (15.2)	87 (20.1)	99 (67.3)	34 (56.7)	65 (74.7)
At least one IADL difficulty	488 (59.0)	230 (58.4)	258 (59.6)	175 (58.8)	63 (56.1)	86 (61.2)

Note: ^a^ the estimates are based on the respondents with difficulty in each ADL.

**Table 3:** Share of older persons with unmet needs in ADLs by family/household characteristics.

Family/Household Structure	BADLs			IADLs			Any ADLs			Totals
	Males	Females	Both	Males	Females	Both	Males	Females	Both	
Overall estimates	66 (16.8)	98 (22.6)	164 (19.8)	129 (32.7)	158 (36.5)	287 (34.7)	135 (34.3)	167 (38.6)	302 (36.5)	827
Marital status										
Married	40 (14.7)	16 (12.9)	56 (14.1)	85 (31.3)	42 (33.9)	127 (32.1)	88 (32.4)	43 (34.7)	131 (33.1)	396
Single/separated/divorced	10 (20.8)	5 (21.7)	15 (21.1)	16 (33.3)	6 (26.1)	22 (31.0)	18 (37.5)	9 (39.1)	27 (38.0)	71
widowed	16 (21.6)	77 (26.9)	93 (25.8)	28 (37.8)	110 (38.5)	138 (38.3)	29 (39.2)	115 (40.2)	144 (40.0)	360
	3.83	11.92 *	17.13 **	4.31	3.80	6.57	4.54	2.4	5.71	
Family size										
≤4	32 (21.5)	62 (27.2)	94 (24.9)	50 (33.6)	103 (45.2)	153 (40.6)	52 (34.9)	107 (46.9)	159 (42.2)	377
5 or more	34 (13.9)	36 (17.6)	70 (15.6)	79 (32.2)	55 (26.8)	134 (29.8)	83 (33.9)	60 (29.3)	143 (31.8)	450
χ^2^	4.00	5.79	11.35 **	0.08	16.43 ***	10.94**	1.74	14.56 **	9.78 **	
Family type										
Monogamy	36 (15.5)	41 (21.1)	77 (18.1)	83 (35.8)	65 (33.5)	148 (34.7)	84 (36.2)	72 (37.1)	156 (36.6)	426
Polyandry	30 (18.5)	57 (23.9)	87 (21.7)	46 (28.4)	93 (38.9)	139 (34.7)	51 (31.5)	95 (39.8)	146 (36.4)	401
χ^2^	1.06	0.81	1.70	2.87	4.38	2.85	1.83	0.57	0.07	
Household head										
Self	57 (19.0)	72 (28.8)	129 (23.5)	99 (33.0)	99 (39.6)	198 (36.0)	104 (34.7)	105 (42.0)	209 (38.0)	550
Someone else	9 (9.6)	26 (14.2)	35 (12.6)	30 (31.9)	59 (32.2)	89 (32.1)	31 (33.0)	62 (33.9)	93 (33.6)	277
χ^2^	6.95 *	16.00 ***	13.58**	6.24	4.02	1.78	4.11	5.82	1.61	
Household living Arrangements										
Alone	19 (20.7)	31 (28.4)	50 (24.9)	35 (38.0)	42 (38.5)	77 (38.3)	38 (41.3)	47 (43.1)	85 (42.3)	201
With immediate family	37 (14.7)	37 (17.7)	74 (16.1)	75 (29.9)	66 (31.6)	141 (30.7)	78 (31.1)	70 (33.5)	148 (32.2)	460
With others	10 (19.6)	30 (26.1)	40 (24.1)	19 (37.3)	50 (43.5)	69 (41.6)	19 (37.3)	50 (43.5)	69 (41.6)	166
χ^2^	4.82	6.71	12.12 *	3.82	8.29	12.46 *	3.80	6.27	10.09 *	
Proximity to children/caregivers										
Far	14 (16.7)	20 (25.3)	34 (20.9)	27 (32.1)	30 (38.0)	57 (35.0)	28 (33.3)	33 (41.8)	61 (37.4)	163
Close	31 (20.4)	56 (28.0)	87 (24.7)	55 (36.2)	86 (43.0)	141 (40.1)	58 (38.2)	89 (44.5)	147 (41.8)	352
Very close	21 (13.3)	22 (14.3)	43 (13.8)	47 (29.8)	42 (27.3)	89 (28.5)	49 (31.0)	45 (29.2)	94 (30.1)	312
χ^2^	3.40	13.65 **	16.24 **	3.50	14.82 **	16.62 **	3.13	10.63 *	12.27 *	

Note: estimates are based on the total sample of the respondents. χ^2^ Chi-squared statistic. Significance level: * *p* < 0.05, ** *p* < 0.01, *** *p* < 0.001.

**Table 4:** Poisson–logit hurdle regression (PLHR) estimates the effects of family/household structures on the probability of experiencing unmet needs and the number of unmet needs of older adults in Oyo State.

	B-ADL		I-ADL		Any ADL	
	β (95%, C.I.)	SE	β (95%, C.I.)	SE	β (95%, C.I.)	SE
Binomial model (zero hurdle with logit model)						
Current marital status						
Married ^RC^						
Single/separated/divorced	−0.08 (−0.80, 0.63)	0.37	0.34 (−0.27, 0.95)	0.31	0.06 (−0.53, 0.65)	0.30
Widowed	−0.23 (−0.77, 0.30)	0.27	−0.04 (−0.46, 0.39)	0.22	−0.01 (−0.43, 0.41)	0.22
Family size	0.07 (−0.01, 0.15)	0.04	0.05 (−0.01, 0.12)	0.03	0.03 (−0.03, 0.10)	0.03
Household wealth status						
Poor ^RC^						
Moderate	−0.54 (−1.04, −0.04) *	0.26	−0.18 (−0.60, 0.25)	0.22	−0.17 (−0.59, 0.25)	0.22
Rich	0.00 (−0.46, 0.46)	0.23	0.03 (−0.34, 0.40)	0.19	−0.02 (−0.38, 0.35)	0.19
Family type						
Monogamy ^RC^						
Polygamy	0.03 (−0.36, 0.43)	0.20	0.09 (−0.24, 0.41)	0.17	0.11 (−0.21, 0.43)	0.16
Household head						
Self ^RC^						
Someone else	0.68 (0.22, 1.14) **	0.23	0.08 (−0.27, 0.44)	0.18	0.10 (−0.25, 0.45)	0.18
Living arrangements						
Alone ^RC^						
With children/spouse	0.13 (−0.36, 0.62)	0.25	0.23 (−0.17, 0.63)	0.20	0.33 (−0.07, 0.72)	0.20
With others	0.15 (−0.38, 0.67)	0.27	−0.09 (−0.54, 0.35)	0.23	0.10 (−0.35, 0.54)	0.23
Family bond						
Not close ^RC^						
Somewhat close	0.18 (−0.31, 0.67)	0.25	−0.05 (−0.48, 0.38)	0.22	−0.04 (−0.46, 0.38)	0.22
Very close	0.39 (−0.12, 0.90)	0.26	0.09 (−0.35, 0.52)	0.22	0.10 (−0.33, 0.53)	0.22
Caregivers’ socioeconomic status						
Low ^RC^						
Moderate	−0.50 (−1.19, 0.18)	0.35	−0.15 (−0.69, 0.39)	0.27	−0.21 (−0.74, 0.33)	0.27
High	−0.39 (−0.94, 0.17)	0.28	−0.23 (−0.65, 0.20)	0.22	−0.29 (−0.71, 0.13)	0.21
Proximity to immediate family						
Far ^RC^						
Closer/very close	0.46 (0.04, 0.88) *	0.22	0.31 (−0.02, 0.65)	0.17	0.29 (−0.04, 0.62)	0.17
Count model (zero-truncated Poisson regression model)						
Current marital status						
Married ^RC^						
Single/separated/divorced	0.24 (−0.07, 0.55)	0.16	0.18 (−0.09, 0.45)	0.14	0.16 (−0.03, 0.36)	0.10
widowed	0.14 (−0.10, 0.38)	0.12	0.24 (0.04, 0.44) *	0.10	0.27 (0.13, 0.42) ***	0.07
Family size	−0.04 (−0.08, 0.00) *	0.02	−0.03 (−0.06, 0.00)	0.02	−0.06 (−0.08, −0.03) **	0.01
Household wealth status						
Poor ^RC^						
Moderate	−0.13 (−0.34, 0.08)	0.11	0.11 (−0.07, 0.29)	0.09	0.13 (0.00, 0.26)	0.07
Rich	−0.33 (−0.54, −0.12) **	0.11	−0.26 (−0.44, −0.09) **	0.09	−0.29 (−0.42, −0.17) ***	0.06
Family type						
Monogamy ^RC^						
Polygamy	0.19 (0.01, 0.37) *	0.09	−0.02 (−0.17, 0.13)	0.08	0.06 (−0.04, 0.17)	0.06
Household head						
Self ^RC^						
Someone else	−0.03 (−0.25, 0.19)	0.11	−0.22 (−0.40, −0.05) *	0.09	−0.27 (−0.40, −0.15) ***	0.07
Living arrangements						
Alone ^RC^						
With children/spouse	0.09 (−0.13, 0.32)	0.11	0.05 (−0.13, 0.23)	0.09	0.12 (−0.01, 0.25)	0.07
With others	0.31 (0.09, 0.52) **	0.11	0.06 (−0.12, 0.25)	0.09	0.19 (0.05, 0.32) **	0.07
Family bond						
Not close ^RC^						
somewhat close	−0.07 (−0.29, 0.16)	0.11	−0.16 (−0.34, 0.03)	0.10	−0.18 (−0.31, −0.04) *	0.07
Very close	−0.26 (−0.49, −0.03) *	0.12	−0.24 (−0.44, −0.05) *	0.10	−0.31 (−0.45, −0.17) ***	0.07
Caregivers’ socioeconomic status						
Low ^RC^						
Moderate	0.10 (−0.22, 0.42)	0.16	0.32 (0.06, 0.58) *	0.13	0.32 (0.13, 0.51) **	0.10
High	0.03 (−0.23, 0.28)	0.13	0.17 (−0.04, 0.38)	0.11	0.17 (0.01, 0.32) *	0.08
Proximity to immediate family						
Far ^RC^						
Closer/very close	−0.14 (−0.34, 0.07)	0.10	−0.22 (−0.39, −0.05) *	0.09	−0.19 (−0.32, −0.07) **	0.06
Model diagnosis						
Log likelihood	−768.9254		−1090.9088		−1536.6122	
Wald chi2 (Prob > chi2)	69.63 (*p* < 0.001)		55.08 (*p* < 0.001)		54.59 (*p* < 0.001)	
AIC	1.918		2.696		3.774	

Note: β: adjusted regression coefficient, adjusting for sociodemographic variables as covariates including sex, age, level of education, religion, economic activity status, and sources of income. C.I. confidence interval; SE standard error. RC reference category; Significance level: * *p* < 0.05; ** *p* < 0.01; *** *p* < 0.001.

## Data Availability

The data presented in this study are available on request from the author. The data are not publicly available due to the confidential information of the respondents in the dataset.

## Appendix A

**Table A1:** Multicollinearity test using variance inflation factor (VIF).

Variable	VIF	1/VIF
Family bond (very close)	2.13	0.469477
Marital status (widow)	2.02	0.495511
Family bond (somewhat close)	1.87	0.534231
Income source (family/children)	1.84	0.544047
Age (80+)	1.83	0.547862
Living with son/daughter	1.80	0.556839
Caregivers’ SES (high)	1.75	0.572821
Economic activity (engaged)	1.69	0.592122
Caregivers’ SES (moderate)	1.64	0.608866
Sex (female)	1.63	0.612539
Household wealth status (high)	1.58	0.634004
Living with non-family	1.54	0.647873
Household wealth status (moderate)	1.50	0.665571
Age (70–79)	1.45	0.689325
Level of education (primary)	1.39	0.717701
Level of education (secondary)	1.36	0.732835
Marital status (single/divorced/separated)	1.27	0.789114
Household head (someone else)	1.23	0.811021
Number of children alive	1.21	0.823500
Family type (polygamy)	1.20	0.831290
Income source (both self and family)	1.17	0.852974
Religion (non-Christian)	1.16	0.859719
Proximity to children (close/very close)	1.13	0.885366
Mean VIF	1.54	

Note: A dummy of each categorical variable was used. The variables are arranged in order of the VIF estimates.

**Table A2:** Poisson–logit hurdle regression (PLHR) estimates the effects of family/household structures on the probability of experiencing unmet needs and the number of unmet needs of older adults in Oyo State.

	BADL		IADL		Any ADL	
	β (95%, C.I.)	SE	β (95%, C.I.)	SE	β (95%, C.I.)	SE
Binomial model (zero hurdle with logit model)						
Marital status						
Married ^RC^						
Single/separated/divorced	−0.08 (−0.80, 0.63)	0.37	0.34 (−0.27, 0.95)	0.31	0.06 (−0.53, 0.65)	0.30
Widowed	−0.23 (−0.77, 0.30)	0.27	−0.04 (−0.46, 0.39)	0.22	−0.01 (−0.43, 0.41)	0.22
Family size	0.07 (−0.01, 0.15)	0.04	0.05 (−0.01, 0.12)	0.03	0.03 (−0.03, 0.10)	0.03
Household wealth status						
Poor ^RC^						
Moderate	−0.54 (−1.04, −0.04) *	0.26	−0.18 (−0.60, 0.25)	0.22	−0.17 (−0.59, 0.25)	0.22
Rich	0.00 (−0.46, 0.46)	0.23	0.03 (−0.34, 0.40)	0.19	−0.02 (−0.38, 0.35)	0.19
Family type						
Monogamy ^RC^						
Polyandry	0.03 (−0.36, 0.43)	0.20	0.09 (−0.24, 0.41)	0.17	0.11 (−0.21, 0.43)	0.16
Household head						
Self ^RC^						
Someone else	0.68 (0.22, 1.14) **	0.23	0.08 (−0.27, 0.44)	0.18	0.10 (−0.25, 0.45)	0.18
Living arrangements						
Alone ^RC^						
With children/spouse	0.13 (−0.36, 0.62)	0.25	0.23 (−0.17, 0.63)	0.20	0.33 (−0.07, 0.72)	0.20
With others	0.15 (−0.38, 0.67)	0.27	−0.09 (−0.54, 0.35)	0.23	0.10 (−0.35, 0.54)	0.23
Family bond						
Not close ^RC^						
Somewhat close	0.18 (−0.31, 0.67)	0.25	−0.05 (−0.48, 0.38)	0.22	−0.04 (−0.46, 0.38)	0.22
Very close	0.39 (−0.12, 0.90)	0.26	0.09 (−0.35, 0.52)	0.22	0.10 (−0.33, 0.53)	0.22
Caregivers’ socioeconomic status						
Low ^RC^						
Moderate	−0.50 (−1.19, 0.18)	0.35	−0.15 (−0.69, 0.39)	0.27	−0.21 (−0.74, 0.33)	0.27
High	−0.39 (−0.94, 0.17)	0.28	−0.23 (−0.65, 0.20)	0.22	−0.29 (−0.71, 0.13)	0.21
Proximity to immediate family						
Far ^RC^						
Closer/very close	0.46 (0.04, 0.88) *	0.22	0.31 (−0.02, 0.65)	0.17	0.29 (−0.04, 0.62)	0.17
Sex						
Male ^RC^						
Female	−0.19 (−0.67, 0.29)	0.24	−0.02 (−0.40, 0.36)	0.19	−0.08 (−0.46, 0.30)	0.19
Age						
<70 ^RC^						
70–79	0.15 (−0.30, 0.61)	0.23	0.08 (−0.29, 0.45)	0.19	0.06 (−0.31, 0.43)	0.19
80+	0.26 (−0.26, 0.79)	0.27	0.26 (−0.19, 0.70)	0.23	0.30 (−0.14, 0.73)	0.22
Religion						
Christianity ^RC^						
Others	−0.25 (−0.65, 0.14)	0.20	0.00 (−0.33, 0.33)	0.17	0.01 (−0.32, 0.33)	0.17
Level of education						
None ^RC^						
Primary	0.16 (−0.29, 0.62)	0.23	−0.32 (−0.69, 0.05)	0.19	−0.22 (−0.58, 0.15)	0.19
Secondary/higher	−0.10 (−0.67, 0.47)	0.29	−0.37 (−0.84, 0.10)	0.24	−0.21 (−0.67, 0.26)	0.24
Economic activity						
Not engaged ^RC^						
Engaged	−0.30 (−0.77, 0.17)	0.24	−0.41 (−0.82, −0.01) *	0.21	−0.30 (−0.70, 0.10)	0.20
Income source						
Self-employment/pension ^RC^						
Children/family	−0.87 (−1.37, −0.37) **	0.25	−0.94 (−1.37, −0.52) ***	0.22	−0.93 (−1.35, −0.51) ***	0.21
Both self and family	−1.27 (−1.88, −0.66) ***	0.31	−1.25 (−1.81, −0.69) ***	0.29	−1.26 (−1.82, −0.70) ***	0.28
Count model (zero-truncated Poisson regression model)						
Marital status						
Married ^RC^						
Single/separated/divorced	0.24 (−0.07, 0.55)	0.16	0.18 (−0.09, 0.45)	0.14	0.16 (−0.03, 0.36)	0.10
Widowed	0.14 (−0.10, 0.38)	0.12	0.24 (0.04, 0.44) *	0.10	0.27 (0.13, 0.42) ***	0.07
Family size	−0.04 (−0.08, 0.00) *	0.02	−0.03 (−0.06, 0.00)	0.02	−0.06 (−0.08, −0.03) **	0.01
Household wealth status						
Poor ^RC^						
Moderate	−0.13 (−0.34, 0.08)	0.11	0.11 (−0.07, 0.29)	0.09	0.13 (0.00, 0.26)	0.07
Rich	−0.33 (−0.54, −0.12) **	0.11	−0.26 (−0.44, −0.09) **	0.09	−0.29 (−0.42, −0.17) ***	0.06
Family type						
Monogamy ^RC^						
Polyandry	0.19 (0.01, 0.37) *	0.09	−0.02 (−0.17, 0.13)	0.08	0.06 (−0.04, 0.17)	0.06
Household head						
Self ^RC^						
Someone else	−0.03 (−0.25, 0.19)	0.11	−0.22 (−0.40, −0.05) *	0.09	−0.27 (−0.40, −0.15) ***	0.07
Living arrangements						
Alone ^RC^						
With children/spouse	0.09 (−0.13, 0.32)	0.11	0.05 (−0.13, 0.23)	0.09	0.12 (−0.01, 0.25)	0.07
With others	0.31 (0.09, 0.52) **	0.11	0.06 (−0.12, 0.25)	0.09	0.19 (0.05, 0.32) **	0.07
Family bond						
Not close ^RC^						
Somewhat close	−0.07 (−0.29, 0.16)	0.11	−0.16 (−0.34, 0.03)	0.10	−0.18 (−0.31, −0.04) *	0.07
Very close	−0.26 (−0.49, −0.03) *	0.12	−0.24 (−0.44, −0.05) *	0.10	−0.31 (−0.45, −0.17) ***	0.07
Caregivers’ socioeconomic status						
Low ^RC^						
Moderate	0.10 (−0.22, 0.42)	0.16	0.32 (0.06, 0.58) *	0.13	0.32 (0.13, 0.51) **	0.10
High	0.03 (−0.23, 0.28)	0.13	0.17 (−0.04, 0.38)	0.11	0.17 (0.01, 0.32) *	0.08
Proximity to immediate family						
Far ^RC^						
Closer/very close	−0.14 (−0.34, 0.07)	0.10	−0.22 (−0.39, −0.05) *	0.09	−0.19 (−0.32, −0.07) **	0.06
Sex						
Male ^RC^						
Female	0.19 (−0.04, 0.42)	0.12	0.12 (−0.06, 0.31)	0.09	0.11 (−0.03, 0.24)	0.07
Age						
<70 ^RC^						
70–79	0.21 (0.00, 0.42)	0.11	0.01 (−0.16, 0.17)	0.09	0.07 (−0.06, 0.19)	0.06
80+	0.06 (−0.19, 0.31)	0.13	−0.13 (−0.35, 0.08)	0.11	0.00 (−0.15, 0.15)	0.08
Religion						
Christianity ^RC^						
Others	0.21 (0.03, 0.40) *	0.09	0.09 (−0.05, 0.24)	0.08	0.19 (0.08, 0.30) ***	0.05
Level of education						
None ^RC^						
Primary	0.21 (0.00, 0.41) *	0.10	−0.04 (−0.21, 0.12)	0.08	−0.01 (−0.13, 0.11)	0.06
Secondary/higher	0.43 (0.18, 0.69) **	0.13	0.27 (0.08, 0.47) **	0.10	0.32 (0.18, 0.47) ***	0.07
Economic activity						
Not engaged ^RC^						
Engaged	0.12 (−0.08, 0.32)	0.10	0.13 (−0.04, 0.30)	0.09	0.17 (0.05, 0.30) **	0.06
Income source						
Self-employment/ pension ^RC^						
Children/family	−0.06 (−0.27, 0.15)	0.11	0.11 (−0.07, 0.28)	0.09	0.06 (−0.07, 0.18)	0.07
Both self and family	0.10 (−0.14, 0.34)	0.12	0.24 (0.03, 0.44) *	0.10	0.26 (0.11, 0.41) **	0.08
Model Diagnosis						
Log likelihood	−768.9254		−1090.9088		−1536.6122	
Wald chi2 (Prob > chi2)	69.63 (*p* < 0.001)		55.08 (*p* < 0.001)		54.59 (*p* < 0.001)	
AIC	1.918		2.696		3.774	

Note: β: adjusted regression coefficient, adjusting for sociodemographic variables as covariates including sex, age, level of education, religion, economic activity status, and sources of income. C.I. confidence interval; SE standard error; RC reference category; Significance level:* *p* < 0.05; ** *p* < 0.01; *** *p* < 0.001.
